# Catastrophic Early Hemorrhage During Total Knee Arthroplasty in a Patient With Klippel-Trénaunay Syndrome: A Case Report

**DOI:** 10.7759/cureus.105727

**Published:** 2026-03-23

**Authors:** Kazuya Yashiro, Atsushi Miyazaki, Norihiko Obata

**Affiliations:** 1 Department of Anesthesiology, Kobe University Hospital, Kobe, JPN

**Keywords:** coagulopathy, klippel–trénaunay syndrome, massive intraoperative hemorrhage, total knee arthroplasty, venous malformation

## Abstract

Klippel-Trénaunay syndrome (KTS) is associated with complex venous malformations and a risk of massive intraoperative bleeding. We report a young woman with KTS who developed 2,710 mL of intraoperative bleeding during total knee arthroplasty despite prior sclerotherapy and tourniquet use, requiring postoperative ICU management for coagulopathy. Preoperative MRI demonstrated both extensive vascular involvement and intra-articular venous malformations. This case highlights the importance of preoperative risk assessment based on lesion extent and intra-articular involvement and suggests that hybrid operating room strategies may be considered in selected high-risk cases.

## Introduction

Klippel-Trénaunay syndrome (KTS) is a rare congenital vascular disorder characterized by the triad of capillary malformations, venous anomalies, and hypertrophy of soft tissue and bone [1,2]. Surgical procedures in patients with KTS are challenging, and hemorrhagic complications have been reported in various operative settings [3].

Total knee arthroplasty (TKA) may be required in patients with KTS because of joint destruction caused by recurrent hemarthrosis and hypertrophic osteoarthritis [4-6]. Recent studies suggest that, with modern blood management strategies and careful multidisciplinary planning, TKA can be performed with acceptable outcomes in selected patients with KTS [7]. However, these reports mainly describe total perioperative blood loss and provide little information on the early intraoperative course or anesthetic implications.

In routine practice, TKA performed under tourniquet control is generally considered to provide a nearly bloodless surgical field and forms the basis of anesthetic and transfusion planning. In patients with KTS, however, extensive abnormal venous networks may bypass tourniquet occlusion, rendering this strategy ineffective [3-6].

We report a patient with KTS who developed uncontrollable massive hemorrhage of 1,220 mL within 15 minutes after skin incision during TKA, despite tourniquet use. This case highlights the early failure of standard hemostatic strategies and underscores the need for preemptive preparation for immediate hemodynamic collapse and massive transfusion.

## Case presentation

**Figure 1:**
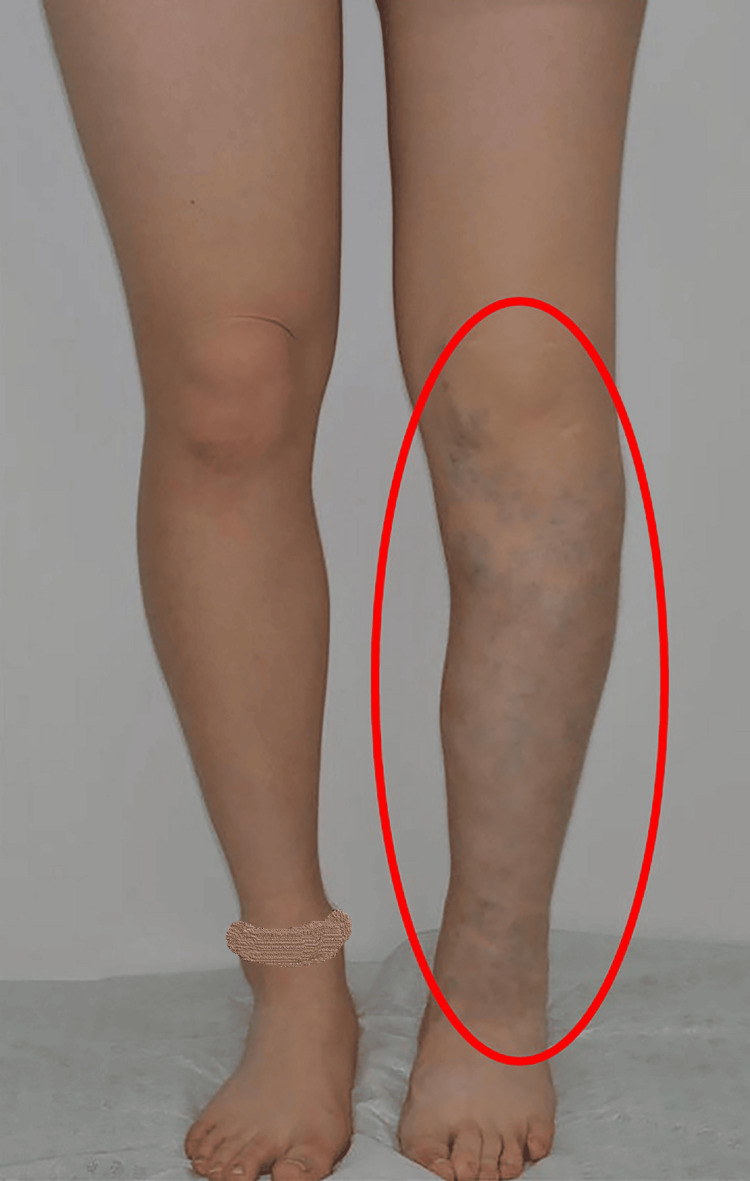
The patient's lower limbs. Abnormal vessels are visible through the subcutaneous tissue (red circle).

A woman in her twenties (height 159 cm, weight 49 kg) had a bluish capillary malformation on her left lower extremity since birth and had been clinically diagnosed with KTS (Figure 1).

**Figure 2:**
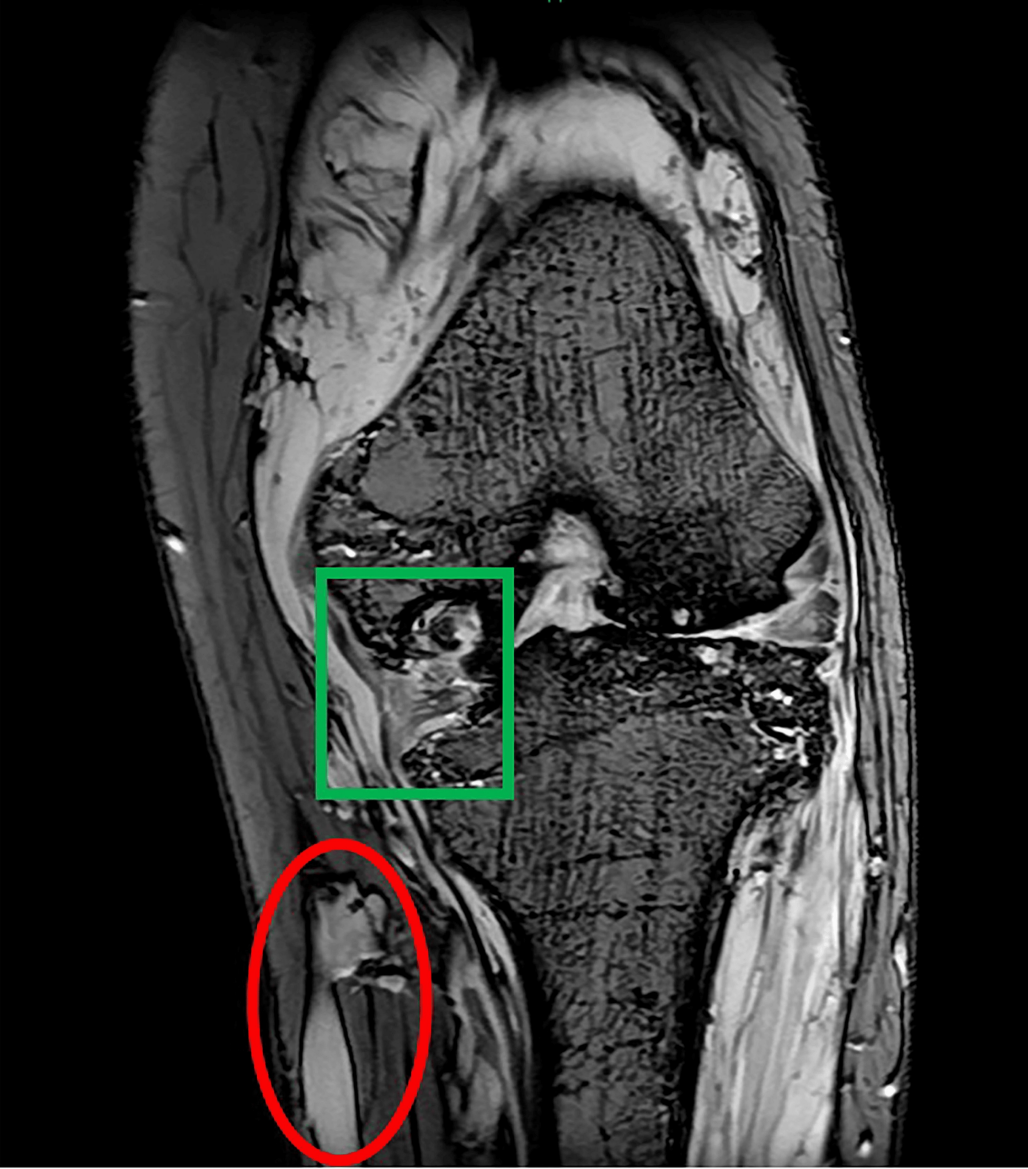
Preoperative T2*-weighted MRI findings. Enlarged veins and varicose veins (red circle) are visible, along with an arteriovenous malformation within the joint (green square). MRI, magnetic resonance imaging

In the outpatient setting, she had been followed by the plastic surgery department and had undergone multiple sessions of sclerotherapy in addition to receiving sirolimus treatment. Sclerotherapy for superficial venous malformations of the left lower limb was performed seven times since childhood. The last sclerotherapy session was performed eight years ago. Following these treatments, the patient underwent regular follow-up visits in the plastic surgery department, including coagulation function testing and imaging evaluations. She gradually developed left knee pain, and magnetic resonance imaging revealed destruction of the medial tibial plateau, injury of the medial collateral ligament, and extensive collateral vessels extending from the subcutaneous tissue into the intra-articular space (Figure 2).

She had no other medical history. Preoperative laboratory findings showed decreased hemoglobin levels and elevated D-dimer and fibrin/fibrinogen degradation products (FDPs) (Table 1).

**Table 1:** Laboratory findings. This table summarizes perioperative changes in laboratory findings obtained preoperatively, after surgery, and on postoperative day 1.

Laboratory parameter	Before surgery	After surgery	Postoperative day 1	Reference range
Hemoglobin (g/dL)	12.2	9.9	6.3	13.7-16.8
Platelet count (×10⁴/μL)	23.3	10.4	5.1	15.8-34.8
Fibrinogen (mg/dL)	261	58	124	200-400
D-dimer (μg/mL)	4.3	16.5	16.6	<1.0
Fibrin degradation product (FDP) (μg/mL)	18.1	61.8	27.9	<5.0
Activated partial thromboplastin time (APTT) (seconds)	28	68.3	27.9	25-38
Prothrombin time (PT) (%)	104	34.6	80	79-116
International normalized ratio (PT-INR)	0.97	1.93	1.13	0.90-1.14

At the time of surgery, general anesthesia was induced and maintained with total intravenous anesthesia using propofol. Tranexamic acid was not administered. After application of a pneumatic tourniquet to the left thigh at 250 mmHg, surgery was initiated.

Bleeding occurred immediately after skin incision. Local hemostatic measures, including electrocautery-based coagulation and ligation, were ineffective. The surgical team judged that the most effective means of hemostasis was compression achieved by tissue approximation and layered closure. At this point, the team was forced to decide whether to abort or proceed with the operation. Because the planned operative time was relatively short (approximately 1 hour), the decision was made to continue the procedure while attempting to control bleeding as much as possible.

Within 15 minutes after the start of surgery, blood loss reached 1,220 mL. At that time, the patient’s vital signs showed a mean arterial pressure (MAP) of 67 mmHg and a heart rate (HR) of 80 bpm. Additional peripheral venous access and an arterial line were established, and a cell salvage system was prepared. Simultaneously, aggressive fluid resuscitation and blood transfusion were initiated. With these interventions, the patient’s hemodynamic status gradually stabilized.

After completion of the arthroplasty, each tissue layer was closed carefully to achieve compression hemostasis. Following skin closure, the tourniquet was gradually released. No obvious bleeding or progressive swelling of the operative field was observed, and the surgery was completed. The total operative time was 1 h 28 min. Total blood loss was 2,710 mL. The patient received 4,500 mL of crystalloid solution and 6 units of packed red blood cells. At the end of surgery, the patient’s vital signs showed a MAP of 61 mmHg, a HR of 120 bpm, and a lactate level of 2.5 mmol/L.

The patient was transferred to the intensive care unit (ICU) under mechanical ventilation. Laboratory findings showed decreased hemoglobin and platelet counts, marked coagulopathy with hypofibrinogenemia, and markedly elevated FDP and D-dimer levels (Table 1). Dilutional coagulopathy or disseminated intravascular coagulation (DIC) was suspected, and 6 units of fresh frozen plasma were transfused.

Hemodynamic stability was subsequently achieved. No persistent bleeding or progression of anemia was observed, and the patient was extubated six hours after ICU admission. On postoperative day 1, hemoglobin levels, platelet counts, and FDP decreased (Table 1). There was no evidence of postoperative bleeding, and the patient was discharged from the ICU. She underwent rehabilitation without rebleeding and was discharged from the hospital on postoperative day 24.

## Discussion

This case demonstrates that catastrophic early failure of hemostasis can occur during TKA in patients with KTS, despite the use of a tourniquet. TKA performed under tourniquet control is generally regarded as a low-bleeding procedure, and anesthetic planning is typically based on the assumption of a nearly bloodless surgical field [8]. In the present case, however, uncontrollable bleeding began immediately after skin incision and progressed rapidly, reaching 1,220 mL within 15 minutes. This temporal pattern suggests that, in KTS, extensive abnormal venous networks may bypass tourniquet occlusion and render standard hemostatic strategies ineffective.

Labott et al. reported a median intraoperative blood loss of 200-275 mL in patients with KTS undergoing TKA and concluded that, with appropriate blood management and careful multidisciplinary perioperative planning, TKA can be performed safely in selected patients [7]. However, their study included one patient who experienced massive blood loss of approximately 4,000 mL. Vascular malformations extending into the intra-articular space in KTS have been identified as a high-risk factor for intraoperative hemorrhage, and substantial bleeding may still occur despite tourniquet application in these cases [6,7]. Furthermore, Barbara and Wilson, in a retrospective analysis of 136 patients with KTS, reported that approximately 4% of TKA procedures were complicated by massive intraoperative hemorrhage [3]. In their study, extensive limb involvement was a common feature among patients who developed severe bleeding.

In the present case, preoperative evaluation revealed intra-articular vascular malformations and superficial venous abnormalities, indicating a high risk of hemorrhage. These findings suggest that massive bleeding may occur immediately after skin incision, even under tourniquet control, and that abnormal venous networks may negate the expected hemostatic effect. From an anesthetic perspective, perioperative planning in such patients should proceed on the assumption that catastrophic hemorrhage may occur. Specifically, large-bore peripheral venous access should be secured before incision, and blood products should be prepared beyond standard practice, including not only packed red blood cells but also fresh frozen plasma and platelet concentrate when indicated by preoperative laboratory findings.

When multiple superficial vessels are concentrated along the planned skin incision line, preoperative sclerotherapy performed at least six weeks before surgery has been recommended to allow sufficient skin healing and to reduce intraoperative bleeding risk [7]. In the present case, multiple sessions of sclerotherapy had already been performed, and the additional benefit of further sclerotherapy to reduce the risk of catastrophic early hemorrhage may be limited.

In the previous studies, preoperative angiographic evaluation was useful for a more precise assessment of the vascular malformations. If feeding vessels had been identified, preoperative embolization might have reduced the risk of massive intraoperative bleeding. Moreover, such imaging findings could have supported a more detailed preoperative discussion with the surgical team regarding the operative strategy, including criteria for aborting the procedure in the event of uncontrolled hemorrhage.

In this case, because the patient had already undergone sclerotherapy for KTS, massive hemorrhage was not strongly anticipated preoperatively. Consequently, only a single intravenous line was secured at the start of surgery, and preparation for rapid transfusion was insufficient. As a result, although balanced transfusion, including fresh frozen plasma and platelet concentrates, might have been considered, only red blood cells were administered intraoperatively. This likely contributed to the marked decrease in fibrinogen to 58 mg/dL after ICU admission.

KTS has also been reported to be associated with Kasabach-Merritt-like consumptive coagulopathy and may progress to DIC [1,3]. Although dilutional coagulopathy was considered the primary mechanism in the present case, the possibility that disease-related DIC also contributed cannot be excluded. Therefore, perioperative management in patients with KTS should account not only for massive hemorrhage but also for the potential development of coagulopathy.

In recent years, hybrid operating rooms equipped to perform intraoperative angiography have become available in some institutions and are frequently utilized in cardiovascular and obstetric surgeries [9,10]. In selected high-risk cases, the use of a hybrid operating room may be considered. Angiographic evaluation before surgery may help identify the vessels that feed vascular malformations, and an embolization could be performed in the same setting if necessary. Although no reports have described the use of a hybrid operating room during TKA in patients with KTS, this strategy may represent a useful option in selected cases, particularly when catastrophic bleeding is strongly anticipated.

## Conclusions

Early catastrophic bleeding can occur during TKA in patients with KTS, even under tourniquet control. Thorough preoperative assessment and readiness for rapid hemostatic intervention, including consideration of a hybrid operating room, are crucial in high-risk cases, particularly in patients with intra-articular vascular abnormalities.
